# The Hyperglycemic Effect of Melatonin in the Chinese Mitten Crab, *Eriocheir sinensis*

**DOI:** 10.3389/fphys.2018.00270

**Published:** 2018-03-21

**Authors:** Xiaozhen Yang, Minjie Xu, Genyong Huang, Cong Zhang, Yangyang Pang, Zhigang Yang, Yongxu Cheng

**Affiliations:** ^1^Key Laboratory of Freshwater Aquatic Resources, Ministry of Agriculture, Shanghai Ocean University, Shanghai, China; ^2^Shanghai Engineering Research Center of Aquaculture, National Demonstration Center for Experimental Fisheries Science Education, Shanghai Ocean University, Shanghai, China; ^3^National Demonstration Center for Experimental Fisheries Science Education, Shanghai Ocean University, Shanghai, China

**Keywords:** melatonin, glucose, eyestalk, crustacean hyperglycemic hormone, *Eriocheir sinensis*

## Abstract

Melatonin has been identified in a variety of invertebrate species, but its function is not as well understood as in crustaceans. The effects of melatonin on hemolymph glucose levels and tissue carbohydrate metabolism in the Chinese mitten crab, *Eriocheir sinensis*, were fully investigated in this study. Moreover, whether the eyestalk (an important endocrine center in invertebrate species) involves in this process or not, also were clarified. Analysis revealed that eyestalk ablation, especially bilateral, caused a significant decrease in the hemolymph glucose level. Moreover, injection of melatonin induced hyperglycemia in a dose-dependent manner both in intact and ablated crabs. Based on the expression of *CHH* mRNA in the 10 different tissues, eyestalk, thoracic ganglion, intestinal tract and hemolymph were selected to estimate the effect of melatonin on the expression of *CHH* mRNA. Bilateral eyestalk ablation caused a significant increase in the expression of *CHH* mRNA in the thoracic ganglion, intestinal tract and hemolymph compared with the controls. In addition, injection of melatonin into intact or ablated crabs elevated the *CHH* mRNA level in the eyestalk, thoracic ganglion and intestinal tract tissues compared with controls. The hemolymph *CHH* mRNA after melatonin injection was elevated only in ablated crabs. Administration of melatonin resulted in a significant decrease in total carbohydrates and glycogen levels with an increase in phosphorylase activity levels in the hepatopancreas and muscle in intact and ablated crabs. Our findings demonstrated that melatonin can induce hyperglycemic effects in both intact and ablated crabs, suggesting that this effect is probably not mediated solely via eyestalk.

## Introduction

Melatonin (5-methoxy-N-acetyltryptamine, MT), is a highly conserved and ubiquitous molecule present from unicellular organisms to mammals. In vertebrate species, circulating plasma MT is synthesized principally in the pineal gland (Hardeland et al., 2011; Erren and Reiter, 2015; Mizutani et al., 2016), whereas it is mainly released from the optic ganglia of eyestalk in invertebrate species (Maciel F. E. et al., 2008). Moreover, it has been shown that it can also be released from peripheral tissues such as the cerebroid ganglion (Mendoza-Vargas et al., 2017), intestine (Lardone et al., 2014) and hemolymph (Mendoza-Vargas et al., 2017) in crustaceans (Balzer et al., 1997; Tilden et al., 1997). As an established neurotransmitter in animals, MT has caught the attention of crustacean physiologists in recent years because it regulates a broad range of body functions (Sainath et al., 2013). These functions include limb regeneration (Tilden et al., 1997), circadian rhythms (Mendoza-Vargas et al., 2017), the anti-oxidant defense system (Geihs et al., 2016) and reproduction (Girish et al., 2015). Moreover, melatonin can also affect the level of hemolymph glucose (Sainath and Reddy, 2010; Maciel et al., 2014).

Glucose is one of the main energy substrates for the general metabolic processes in crustaceans (Nery and Santos, 1993). Several studies have confirmed that circulating glucose levels vary in response to various environmental stresses imposed on the animals, such as temperature (Spicer et al., 1990; Durand et al., 2000; Ridgway et al., 2006), salinity (Santos and Nery, 1987; Spaargaren and Haefner Jr., 1987), hypoxia (Zou et al., 1996; Maciel J. E. S. et al., 2008; da Silva-Castiglioni et al., 2010; Geihs et al., 2013) and autotomy (Yang et al., 2017). Such changes can furtherly bring more serious consequences, including low anti-bacterial ability (Wang et al., 2017), survival reduction (Wanlem et al., 2011) and abnormal behavior (Chen et al., 2011; Sun et al., 2013). Therefore, researchers are paying increasing attention to which factor or mechanism is involved in the regulation of hemolymph glucose level in crustaceans. Biogenic amines, which serve as neuromodulators, participate in the modulation of hemolymph glucose level through different pathways. These biogenic amines include serotonin (5-HT) (Inohara et al., 2015), dopamine (DA) (Camacho-Jiménez et al., 2017), and MT (Sainath and Reddy, 2010).

The sinus gland neuroendocrine complex located in the eyestalk of most crustaceans is the major endocrine center. Crustacean hyperglycemic hormone (CHH), one of the important neurohormones synthesized and released from the X-organ sinus gland complex, is primarily involved in glucose metabolism. Apart from the eyestalk, it has also been identified in other tissues such as the cerebral ganglia, thoracic ganglion, gill, intestinal tract and others (Webster et al., 2012). Kuo et al. (1995) have documented that DA can mimic the action of CHH in inducing hyperglycemia in intact shrimps, but not in bilaterally eyestalk-ablated individuals in tiger shrimps, *Penaeus monodon*. Sathyanandam et al. (2008) found that 5-HT caused a significant hyperglycemia both in intact and bilaterally eyestalk-ablated shrimps, *Fenneropenaeus indicus*. With regard to MT, both above two phenomena exist in different crustaceans.

The following mechanism of CHH action on hemolymph glucose and carbohydrate metabolism has been reported: CHH stimulates the breakdown of tissue carbohydrate (TCHO) and glycogen by activating glycogen phosphorylase in the hepatopancreas and muscle (Fanjul-Moles, 2006). Glycogen phosphorylase exists in two interconvertible forms, “a” and “b,” and the “a” form is physiologically active, whereas “b” is inactive. Glycogen can be degraded by activating the phosphorylase, and then leak into the hemolymph.

The Chinese mitten crab, *Eriocheir sinensis*, is well known as an “excellent aquatic products” and one of the important species for freshwater aquaculture. Its production had increased to approximately 812,103 tons in 2016 (China Fisheries Yearbook, 2017). This study aims to study the effects of MT on the hemolymph glucose levels and carbohydrate metabolism of a freshwater crab, *E. sinensis*, and to verify the hypothesis that MT induces hyperglycemia in *E. sinensis* by stimulating the release of CHH from the X-organ/sinus gland (XO/SG) or other tissues.

## Materials and methods

### Ethics statement

For this research, all animals were handled in accordance with the permits which were established by Animal Experiments Ethics Committee of Shanghai Ocean University for the care and use of laboratory animals.

### Animals

Intact intermolt female crabs (*E. sinensis*) with body weights of 18.51 ± 2.32 g were collected from a commercial farm in Chongming Island (Shanghai, China). They were kept in a circulating system containing thoroughly aerated freshwater and UV-treated PVC tube as a shelter and the acclimation period is 1 week. The crabs were kept in a glass aquarium under a natural photoperiod of 12L:12D (lights on at 07:00h), a temperature of 20°C and a salinity of 20 psu and were fed the basal diet once daily at 19:00 with approximately 2–5% of their total biomass during the test time. Feeding was stopped 1 day before the commencement of the experiment to avoid changes owing to ingestion.

### Eyestalk ablation experiment

This study investigated whether the regulation of hemolymph glucose levels is through the eyestalk. Crabs were divided into three groups (*n* = 10 for each group), which were intact (C), or had unilateral (Unil-ablated) or bilateral eyestalk ablation (Bil-ablated). The ablation was made by cutting off at the base of the peduncle and applying pressure to the wound for 15 s to minimize fluid loss and help healing (Sainz-Hernández et al., 2008). Crabs were used for experimentation 24 h after eyestalk ablation then hemolymph was collected for the determination of glucose levels.

### Dose-dependent and time-course action of melatonin experiment

On the basis of the above results, we designed this study to filtrate the best injection dose of melatonin for intact and ablated crabs. Crabs were randomly assigned to 14 groups, each containing 10 crabs (intact and ablated with 7 groups each). Crabs were injected in the third pereiopod with crustacean saline (NaCl 0.21 M, KCl 13.6 mM, H_3_BO_3_ 8.6 mM, NaOH 4.75 mM, MgSO_4_·7H_2_O 20 mM, pH 7.2) or melatonin in six dosages (10^−12^, 10^−10^, 10^−9^, 10^−8^, 10^−7^, and 10^−6^ mole/crab) in a 20 L volume. Similarly, crabs were used for experimentation 24 h after eyestalk ablation and then hemolymph was sampled 2 h after injection for glucose level analysis (Swetha et al., 2014). Melatonin was purchased from Sinopharm Chemical Reagent Co., Ltd (Shanghai, China) and dissolved in ethanol and diluted with crustacean saline. The melatonin doses were selected on the basis of earlier studies in freshwater crabs (Sainath and Reddy, 2010). All the test solutions were freshly prepared prior to the start of the experiment.

From the above results, a certain concentration of melatonin and different time points (0, 30, 60, 120, 180, and 240 min) were selected and set to explore the time changes in hemolymph glucose level. Hemolymph from each group (*n* = 10) was collected at corresponding time.

### Hemolymph collection and glucose level analysis

Hemolymph was drawn with a sterile 1-mL syringe from the arthrodial membrane of the third pereiopod 24 h after eyestalk ablation, then kept 4°C overnight and centrifugated at 5000 r/min for 15 min to collect supernatant (Komali et al., 2005). The supernatant was mixed with glucose oxidase reagent and generated colored compounds which were measured at 505 nm and quantified against standards by glucose oxidase assay kit (Nanjing Jiancheng Bioengineering Institute).

### Influence of melatonin on *Chh* mRNA expression and tissue carbohydrate (TCHO), glycogen and phosphorylase activity

On the basis of eyestalk ablation and the dose- and time-dependent experiment, four groups (*n* = 10) were set up in this experiment. They were intact (C), intact crabs injected with melatonin (C+MT), eyestalk-ablated crabs (Ablated) and eyestalk-ablated crabs injected with melatonin (Ablated+MT). At first, to obtain information about *CHH* mRNA expression in tissues, 10 different tissue samples (eyestalk, thoracic ganglion, cerebral ganglion, intestinal tract, hepatopancreas, stomach, heart, hemolymph, muscle, and gill) from intact crabs were analyzed. Then, according to the expression of *CHH* mRNA in different tissues, representative tissues were selected to estimate the effect of melatonin injection on the expression of *CHH* mRNA. This study was also designed to determine the effect of melatonin on glucose metabolism-relevant indicators. Hepatopancreas and muscles were immediately sampled and frozen at −80°C prior to the quantification of carbohydrate (TCHO), glycogen and phosphorylase activities. All tissues were collected 2 h after melatonin injection.

### Measurements of *CHH* mRNA expression

RNA was extracted from tissue samples using Trizol Reagent (Invitrogen, USA). The RNA concentration and purity were determined using a QuawellTM Q5000 (USA) at 260 and 280 nm. RNA was measured by 1% (w/v) denaturing formaldehyde agarose gel electrophoresis to examine integrity. Reverse transcription was performed on 500 ng of RNA following the manufacturer's instructions. First-strand cDNA was synthesized from the DNase-treated RNA using PrimeScript™ RT Master Kit (TaKaRa, Japan).

Q-PCR was performed using the 7500 Real-Time PCR System (Life Tech, USA) through SYBR *Premix Ex Taq*™. The primers used were listed in Table 1 and the PCR program utilized was as follows: denaturation at 95°C for 30 s, 5 s at 95°C, 34 s at 60°C followed by 40 cycles and a melt curve at 65°C, 15 s at 95°C, 60 s at 65°C, 30 s at 95°C (Ci et al., 2015). The reaction system was shown in Table 2. Expression levels of each transcript were normalized by comparison with the amount of 18S rRNA.

**Table 1:** Primers for PCR.

**Primer names**	**Primer sequence (5′-3′)**
18S-F	TCCAGTTCGCAGCTTCTTCTT
18S-R	AACATCTAAGGGCATCACAGA
*CHH*-F	GCTACAGCAACCTCGTCTTCCG
*CHH*-R	TTCTTCCTGCCAACCACCC

**Table 2:** The PCR reaction system.

**Reaction system components**	**Volume (μL)**
SYBR premis Ex *Taq* (Tli RNase Plus) (2×)	5.0
PCR forward primer (10 μmol/L)	0.2
PCR reverse primer (10 μmol/L)	0.2
ROX reference Dye II (50×)	0.2
DNA	1.0
dH_2_O	3.4
Total volume	10.0

### Tissue carbohydrate (TCHO), glycogen and phosphorylase activity analysis

TCHO and glycogen levels were estimated following the method of Carroll et al using 10% trichloroacetic acid (TCA) supernatant and an ethanol precipitate of the TCA supernatant (Carroll et al., 1956; Swetha et al., 2014). The anthrone reagent was added to the supernatant, which was centrifuged at 4000 r/min for 10 min, then the mixture was then boiled in a water bath. The cooled contents were measured at 620 nm against a reagent blank.

Phosphorylase activity in the tissues was estimated by the colorimetric determination of inorganic phosphate liberated from glucose-1-phosphated (G-1-P) following the method of Cori et al. (Cori and Cori, 1952; Cori et al., 1955). The reaction mixture was obtained after a treatment series of the tissue homogenate and incubated respectively for 15 and 30 min to measure the activity of phosphorylase “a” and “b” (Nanjing Jiancheng Bioengineering Institute Kit).

### Statistical analysis

All data were presented as means ± S.E. and all statistical analyses were performed using SPSS (16.0.2 for Windows). One-way ANOVA analysis was used for comparison between groups while student *T*-test analysis was for comparison between two groups. The *p* < 0.05 was regarded as statistically significant.

## Results

### Effect of eyestalk ablation on hemolymph glucose level

**Figure 1:**
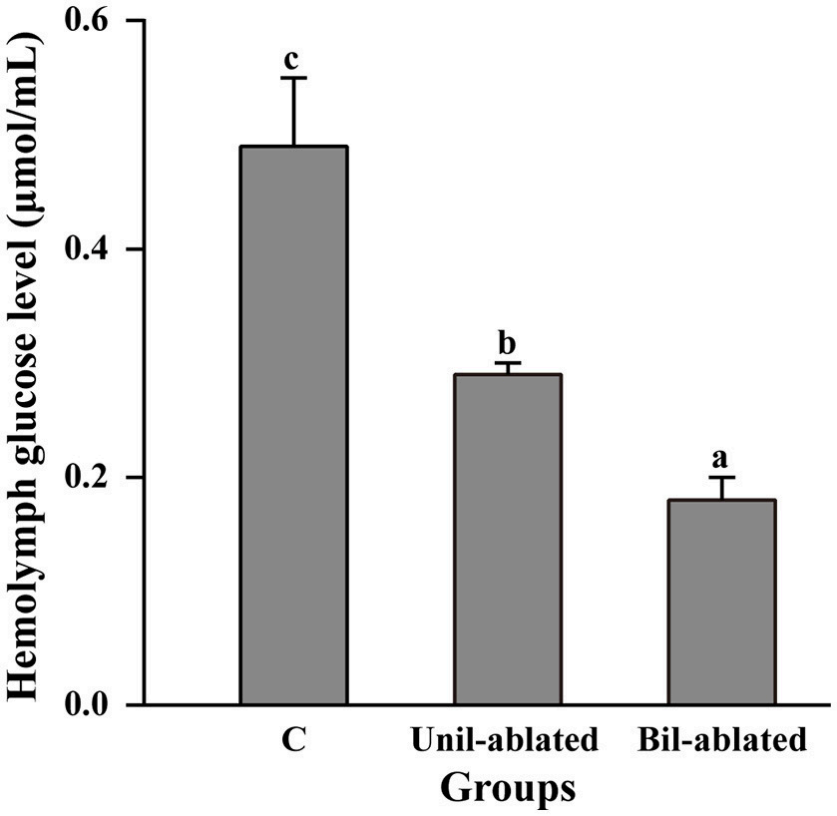
The hemolymph glucose level of *E. sinensis* in intact (C), unilateral (Unil-ablated) and bilateral eyestalk ablated (Bil-ablated) crabs (*n* = 10). Each bar represents a mean ± SE of 10 individual crabs. Values in a row with different lowercase letters denote significant differences (*p* < 0.05).

Whether eyestalk ablation was unilateral or bilateral, it induced a significant decrease in the hemolymph glucose level in the crab *E. sinensis* compared with controls (*p* < 0.05, Figure 1). The hemolymph glucose level in Unil-ablated crabs was significantly higher than in Bil-ablated animals (*p* < 0.05). Therefore, bilateral eyestalk ablation was selected for the subsequent experiments.

### Effect of melatonin on hemolymph glucose level in intact and ablated crabs

**Figure 2:**
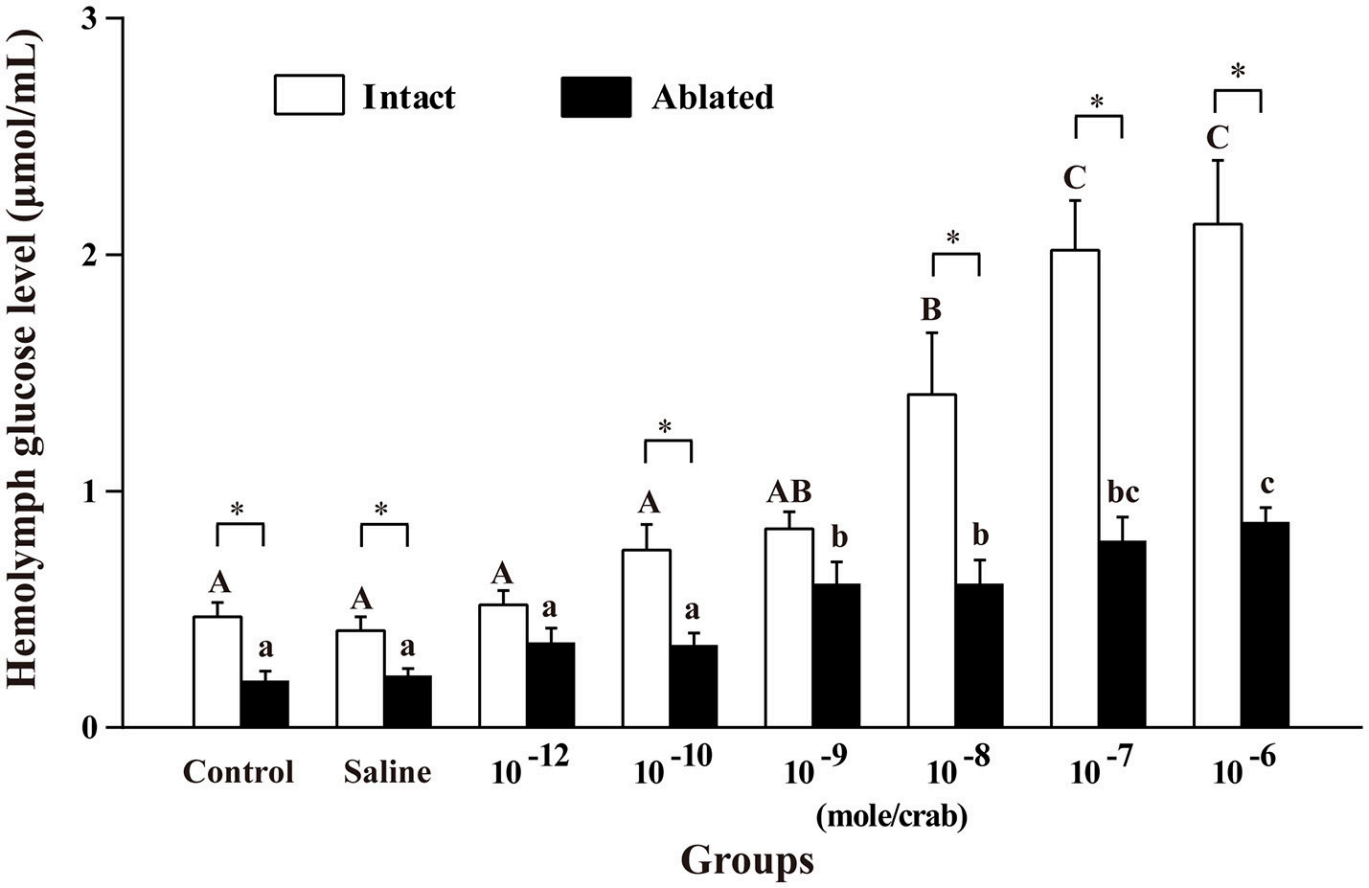
Dose-dependent effects of melatonin on hemolymph glucose level in both intact and ablated crabs, *E. sinensis*. The results are shown as the means ± SE (*n* = 10). The different capital letters in the open bars indicate a significant difference between intact crabs (*p* < 0.05); the different lowercase letters in the solid bars indicate significant differences between ablated crabs (*p* < 0.05). Asterisk (^*^) represents a significant difference (*p* < 0.05) between the intact and ablated groups.

Injection of melatonin into crabs with or without eyestalks resulted in significant hyperglycemia in a dose-dependent manner, whereas injection of crustacean saline did not evoke any significant change in hemolymph glucose levels (*p* > 0.05, Figure 2). At doses of 10^−8^ to 10^−6^ mole/crab, the hyperglycemic effect of intact crabs was statistically significant in a dose-dependent manner compared with the controls (*p* < 0.05). The 10^−6^ mole/crab dose in both intact and ablated crabs was the only a dose that caused a maximal increase in the hemolymph glucose level. Therefore, this dose was used as the test dose in the rest of the experiments.

### Time course of melatonin-induced hyperglycemia

**Figure 3:**
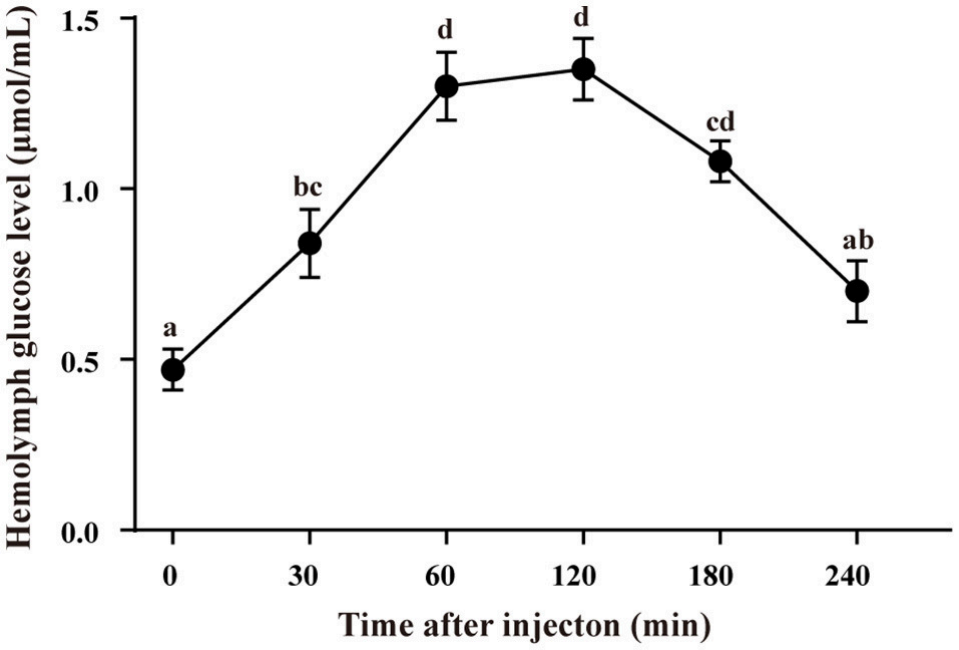
Time course of melatonin-induced hyperglycemia in intact crabs, *E. sinensis*. The results are shown as the means ± SE (*n* = 10). The different lowercase letters indicate significant differences between the groups (*p* < 0.05).

The hemolymph glucose level increased significantly after melatonin injection (10^−6^ mole/crab) and reached the highest peak at 60 and 120 min, after which it went down (Figure 3). There was no significant difference in hemolymph glucose levels between 0 and 240 min (*p* > 0.05).

### Effect of melatonin on the expression of *Chh* mRNA

**Figure 4:**
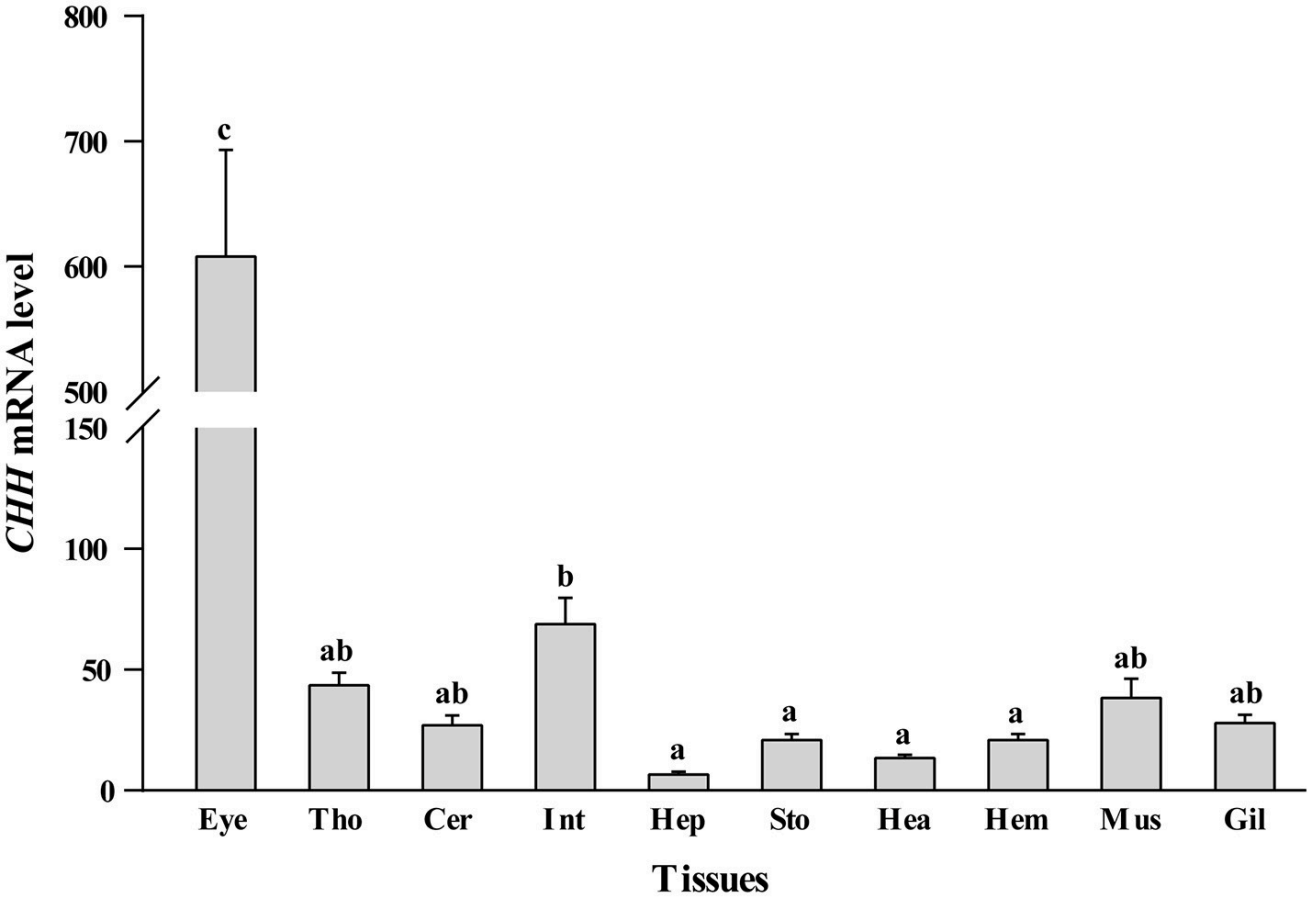
Effect of the injection of melatonin into intact crabs on expression of *CHH* mRNA in tissues. The results are represented as the means ± SE (*n* = 10). Values above a bar with different lowercase letters denote significant differences between the groups (*p* < 0.05). Eye, eyestalk; Tho, thoracic ganglion; Cer, cerebral ganglion; Int, intestinal tract; Hep, hepatopancreas; Sto, stomach; Hea, heart; Hem, hemolymph; Mus, muscle; Gil, gill.

The RT-PCR results showed that the tissue-specific *CHH* mRNA expression was detected in all samples. The level of *CHH* mRNA expression was the highest in the eyestalk and was higher in intestinal tract compared to the other groups (*p* < 0.05, Figure 4). The lowest *CHH* mRNA levels were obtained in the hepatopancreas, stomach, heart and hemolymph, and there was no significant difference in these groups (*p* > 0.05). Moreover, *CHH* mRNA levels were higher in thoracic and cerebral ganglion, muscle and gill than in the lowest group (*p* < 0.05).

**Figure 5:**
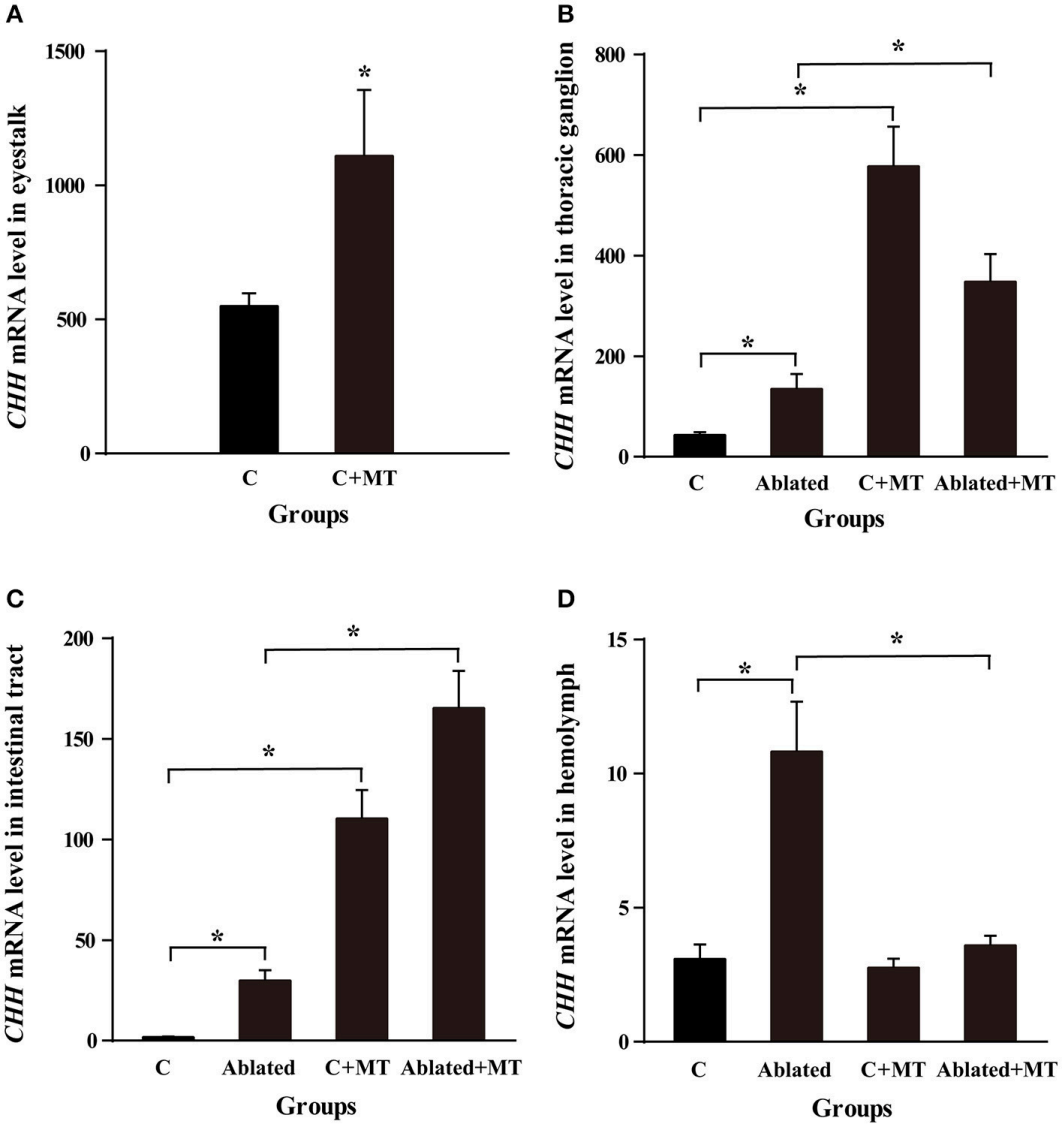
Comparisons of *CHH* mRNA expressions in the eyestalk **(A)**, thoracic ganglion **(B)**, intestinal tract **(C)** and hemolymph **(D)** in intact and, ablated crabs (*E. sinensis*), both injected with melatonin (C+MT and Ablated+MT). Results are shown as the means ± SE (*n* = 10). Asterisk (^*^) represent significant differences between the C and C+MT, C and Ablated, Ablated and Ablated+MT groups.

From the above results, four tissues, the eyestalk, thoracic ganglion, intestinal tract and hemolymph, were selected to estimate the effect of melatonin on the relative expression of *CHH* mRNA. Bilateral eyestalk ablation caused a significant increase in the relative expression of *CHH* mRNA in the thoracic ganglion, intestinal tract and hemolymph compared with controls (*p* < 0.05, Figure 5). In addition, melatonin injection into intact crabs elevated the *CHH* level in the eyestalk, thoracic ganglion and intestinal tract tissues apart from the hemolymph compared with controls (*p* < 0.05). Similarly, melatonin injection into ablated crabs also significantly increased the *CHH* level in the thoracic ganglion and intestinal tract, including also in the hemolymph (*p* < 0.05).

### Effect of melatonin on glucose metabolism

Injection of melatonin into intact crabs resulted in a significant decrease in the TCHO and glycogen levels in the hepatopancreas and muscles compared with the controls (*p* < 0.05, Table 3). In addition, the activity of phosphorylase “a” and “b” were significantly elevated in both the hepatopancreas and muscles of intact crabs (*p* < 0.05, Table 4).

**Table 3:** Effect of melatonin in intact and ablated crabs on hepatopancreas, muscle total carbohydrate (TCHO) and glycogen levels in *E. sinensis*.

**Group**	**TCHO (*n* = 10)**		**Glycogen (*n* = 10)**	
	**Hepatopancreas**	**Muscle**	**Hepatopancreas**	**Muscle**
C	21.50 ± 4.71^b^	14.50 ± 2.07^b^	3.34 ± 0.44^b^	9.18 ± 1.37^b^
C+MT	17.64 ± 5.34^a^	11.08 ± 0.60^a^	2.74 ± 0.15^a^	5.67 ± 1.95^a^
Ablated	28.78 ± 3.19^B*^	20.58 ± 5.13^B*^	7.71 ± 2.94^B*^	8.33 ± 1.95^B^
Ablated+MT	14.57 ± 0.98^A^	14.37 ± 2.19^A^	4.31 ± 0.57^A^	1.37 ± 0.15^A^

The different lowercase letters in the first two lines indicate a significant difference between the C and C+MT groups (p < 0.05); the different capital letters in the last two lines indicate a significant difference between the Ablated and Ablated+MT groups (p < 0.05); Asterisk (^*^) represents a significant difference (p < 0.05) between the C and Ablated groups.

**Table 4:** Effect of melatonin injection into intact and ablated crabs on hepatopancreas and muscle glycogen phosphorylase activity levels in *E. sinensis*.

**Group**	**Hepatopancreas phosphorylase (*n* = 10)**		**Muscle phosphorylase (*n* = 10)**	
	**A**	**b**	**a**	**b**
C	5.59 ± 0.17^a*^	12.34 ± 2.08^a*^	9.58 ± 2.34^a^	10.75 ± 3.94
C+MT	9.85 ± 3.33^b^	28.23 ± 7.75^b^	13.99 ± 3.26^b^	8.13 ± 3.13
Ablated	2.58 ± 0.41^A^	5.87 ± 0.59^A^	7.01 ± 3.25^A^	8.03 ± 2.39^A^
Ablated+MT	15.01 ± 12.07^B^	19.14 ± 3.91^B^	17.20 ± 1.65^B^	12.45 ± 1.11^B^

The different lowercase letters in the first two lines indicate a significant difference between the C and C+MT groups (p < 0.05); the different capital letters in the last two lines indicate a significant difference between the Ablated and Ablated+MT groups (p < 0.05); Asterisk (^*^) indicates a significant difference (p < 0.05) between the C and Ablated groups.

Bilateral eyestalk ablation resulted in a significant increase in the TCHO and glycogen levels and a significant decrease in phosphorylase “a” and “b” in the hepatopancreas (*p* < 0.05). Melatonin injection into ablated crabs caused a significant decrease in the TCHO and glycogen levels and elevated phosphorylase “a” and “b” both in the hepatopancreas and muscles (*p* < 0.05, Tables 3, 4).

## Discussion

In our study, we found that eyestalk can influence the level of hemolymph glucose level in *E. sinensis*. Melatonin can regulate the level of hemolymph glucose level by influenced *CHH* mRNA expression in the thoracic ganglion, intestinal tract and hemolymph, and TCHO and glycogen levels and phosphorylase activity levels in the hepatopancreas and muscle.

As we all known, the eyestalks of decapod crustaceans are important endocrine structure that produce and release a host of regulatory neuropeptides (Soyez, 1997; Pillai et al., 2010; Nithya et al., 2013), which play a central role in the regulation of diverse physiological functions such as molting, reproduction, hemolymph glucose levels and others. Therefore, eyestalk ablation is a classical operation to induce growth or precocious maturation of the gonad in commercial brood production (Sainath and Reddy, 2011). The effects of eyestalk ablation on hemolymph glucose levels have been reported in the crabs, *Neohelice granulata* (Maciel et al., 2014), a freshwater edible crab, *Oziotelphusa senex senex* (Sainath and Reddy, 2010) and the fiddler crab, *Uca pugilator* (Tilden et al., 2001). However, the role of the eyestalk in hemolymph glucose physiology has still not been extensively examined in *E. sinensis*. The present data demonstrate that both unilateral and bilateral eyestalk ablation can induce significantly low hemolymph glucose levels, especially bilateral ablation. These results are in agreement with earlier results in *N. granulata* (Maciel et al., 2014) and *O. senex senex* (Sainath and Reddy, 2010).

Biogenic amines in crustaceans are classical neuroregulators which mediate and control the hemolymph glucose level through different mechanisms (Kuo et al., 1995; Girish et al., 2015; Zarazaga et al., 2017). However, studies referring to 5-HT and DA have yielded conflicting results about the eyestalk modulating hemolymph glucose or not in different species. Many studies showed that both 5-HT and DA induce hyperglycemia through the eyestalk, such as in the freshwater prawn, *Macrobrachium malcolmsonii* (Komali et al., 2005) and the crayfish, *Procambarus clarkii* (Lee et al., 2000, 2001; Zou et al., 2003). To date, opinions increasingly support that DA exerts its modulation on hyperglycemic effects through the eyestalk neurosecretory center, while 5-HT produces hyperglycemia in crustaceans by regulating the eyestalk and other sites. In addition to the above two biogenic amines, MT can also influence the hemolymph glucose levels. However, studies have rarely been carried out on *E. sinensis*. Our findings clearly demonstrate that MT induced hyperglycemia in intact and ablated *E. sinensis* in a dose-dependent manner (Figure 2). This result is in agreement with the observations of Sainath and Reddy in the freshwater edible crab, *O. senex senex* (Sainath and Reddy, 2010); and Tilden et al. (2001) in the fiddler crab, *U. pugilator*. Maciel et al. (2014) also documented that melatonin-induced hyperglycemia in the crab, N. granulate was based on the hypoxia condition, but these responses were eyestalk-dependent. The mechanisms underlying melatonin-induced hyperglycemia can vary among the species and depend upon physiological conditions.

Biogenic amines exert their hyperglycemic effect by stimulating the release of CHH from the eyestalk neurosecretory center (Lorenzon et al., 2005). In contrast to this, previous studies reported that elevation of hemolymph glucose levels via biogenic amines occurs independently of CHH, especially for 5-HT. In the present study, MT increased the thoracic ganglion and intestinal tract *CHH* level with corresponding changes in glucose levels both in the intact and ablated animals and the MT-induced increases in hemolymph *CHH* level only in ablated animals. This is based on the location of CHH in 10 different tissues in this study and similar results from earlier studies. This study is the first concerning the hyperglycemic effect of melatonin not mediated solely via eyestalk factors such as CHH and triggering the release of CHH both from the eyestalk and other tissues, mainly in the ganglion and intestinal tract. CHH exiting from the intestinal tract and regulating the molting of crabs have been demonstrated, but its pathway still is no clear (Chung et al., 1999). Moreover, melatonin-induced hyperglycemia in the crab *N. granulate* after exposure to hypoxia downregulated the *CHH*, suggesting a negative feedback response to the increase in circulation glucose levels (Chung and Zmora, 2008). Further research on the mechanism of the hyperglycemic effect of melatonin in *E. sinensis* is needed.

The results presented in this study suggest that MT is involved in the regulation of carbohydrate metabolism in the freshwater Crab, *E. sinensis*. The effect of eyestalk hormones on tissue carbohydrate levels and phosphorylase activity has been extensively studied in several crustaceans (Ramamurthi et al., 1968; Sagardia, 1969; Webster et al., 2012). This study found that MT leads to a significant decrease in TCHO and glycogen levels with an increase in phosphorylase activity in the hepatopancreas and muscle, whether in intact or ablated crabs. Similar studies can also be found for the freshwater edible crab, *O. senex senex* and the freshwater prawn, *M. malcolmsonii*. These results imply glycogenolysis in these tissues and the mobilization of glucose from the hepatopancreas and muscle into the hemolymph. As is known to all, glucose in the hemolymph can provide the energy which is required for the histiocyte activities in the organism. The imbalance of hemolymph glucose regulation can cause the disorder of glucose metabolism and the accumulation of a large number of metabolites which can lead to abnormal immune function (Li and Ouyang, 2017).

In general, MT produces a hyperglycemic effect on the Chinese Mitten Crab, *E. sinensis*, in a dose-and time-dependent manner. The hyperglycemic effect resulted from CHH release from the eyestalk or other tissues, including the central nervous tissues and peripheral tissues. Glycogenolysis by phosphorylase activity from the hepatopancreas and muscle is involved in this process.

## Conclusion

From the results of this study, we conclude that eyestalk ablation, especially bilateral significantly influences the hemolymph glucose level. Injection of melatonin induced hyperglycemia in a dose-dependent manner both in intact and ablated crabs. *CHH* mRNA expression had tissue-specific characteristic in *E. sinensis*. Moreover, bilateral eyestalk ablation caused a significant increase in the expression of *CHH* mRNA in the thoracic ganglion, intestinal tract and hemolymph. Whether they underwent eyestalk ablation or not, crabs injected with melatonin had a high expression of *CHH* mRNA. Administration of melatonin resulted in a decrease in total carbohydrates (TCHO) and glycogen levels with an increase in phosphorylase activity levels in the hepatopancreas and muscle in intact and ablated crabs.

## Author contributions

XY and MX conceived and designed the experiments; MX performed the experiments and analyzed the data; YC was the project administration and funding acquisitions; GH, CZ, YP, and ZY gave the guidance for the study. All authors read and approved the final manuscript.

### Conflict of interest statement

The authors declare that the research was conducted in the absence of any commercial or financial relationships that could be construed as a potential conflict of interest.
